# Microstructural alterations in white matter and related neurobiology based on the new clinical subtypes of Parkinson's disease

**DOI:** 10.3389/fnins.2024.1439443

**Published:** 2024-08-01

**Authors:** Xiaorong Yuan, Qiaowen Yu, Yanyan Liu, Jinge Chen, Jie Gao, Yujia Liu, Ruxi Song, Yingzhi Zhang, Zhongyu Hou

**Affiliations:** ^1^Department of Medical Imaging, Shandong Provincial Hospital Affiliated to Shandong First Medical University, Jinan, Shandong, China; ^2^Department of Medical Imaging, Shandong Provincial Hospital, Jinan, Shandong, China; ^3^Department of Radiology, Shandong Mental Health Center, Jinan, Shandong, China; ^4^Department of Medical Imaging, Shandong Provincial Third Hospital, Jinan, Shandong, China; ^5^Department of Radiology, Binzhou Medical University Hospital, Binzhou, China

**Keywords:** Parkinson's disease, new clinical subtypes, DTI, longitudinal study, neurobiology

## Abstract

**Background and objectives:**

The advent of new clinical subtyping systems for Parkinson's disease (PD) has led to the classification of patients into distinct groups: mild motor predominant (PD-MMP), intermediate (PD-IM), and diffuse malignant (PD-DM). Our goal was to evaluate the efficacy of diffusion tensor imaging (DTI) in the early diagnosis, assessment of clinical progression, and prediction of prognosis of these PD subtypes. Additionally, we attempted to understand the pathological mechanisms behind white matter damage using single-photon emission computed tomography (SPECT) and cerebrospinal fluid (CSF) analyses.

**Methods:**

We classified 135 de novo PD patients based on new clinical criteria and followed them up after 1 year, along with 45 healthy controls (HCs). We utilized tract-based spatial statistics to assess the microstructural changes of white matter at baseline and employed multiple linear regression to examine the associations between DTI metrics and clinical data at baseline and after follow-up.

**Results:**

Compared to HCs, patients with the PD-DM subtype demonstrated reduced fractional anisotropy (FA), increased axial diffusivity (AD), and elevated radial diffusivity (RD) at baseline. The FA and RD values correlated with the severity of motor symptoms, with RD also linked to cognitive performance. Changes in FA over time were found to be in sync with changes in motor scores and global composite outcome measures. Furthermore, baseline AD values and their rate of change were related to alterations in semantic verbal fluency. We also discovered the relationship between FA values and the levels of α-synuclein and β-amyloid. Reduced dopamine transporter uptake in the left putamen correlated with RD values in superficial white matter, motor symptoms, and autonomic dysfunction at baseline as well as cognitive impairments after 1 year.

**Conclusions:**

The PD-DM subtype is characterized by severe clinical symptoms and a faster progression when compared to the other subtypes. DTI, a well-established technique, facilitates the early identification of white matter damage, elucidates the pathophysiological mechanisms of disease progression, and predicts cognitively related outcomes. The results of SPECT and CSF analyses can be used to explain the specific pattern of white matter damage in patients with the PD-DM subtype.

## Introduction

The clinical manifestations and prognosis of Parkinson's disease (PD) exhibit significant heterogeneity. Identifying distinct PD subtypes is essential for gaining a deeper insight into the mechanisms of disease, enhancing the ability to predict disease progression and outcomes, and ultimately designing more effective personalized care strategies (Fereshtehnejad et al., 2017; Huang and Qian, 2019; Marras et al., 2020; Shakya et al., 2022). Most early PD subtyping protocols are based on a priori hypothesis using a single categorical variable (e.g., age, tremor, etc.), which suffers from inconsistent reliability and confounding disease staging (Fereshtehnejad and Postuma, 2017; Lee et al., 2022). The new clinical subtype is described by a comprehensive set of criteria that summarizes the results of clustering analysis of demographic and genetic information, motor symptoms and signs, neuropsychological assessments, and other nonmotor symptoms. Three distinct subtypes of PD have been defined: mild motor predominant (PD-MMP), intermediate (PD-IM), and diffuse malignant (PD-DM) (Fereshtehnejad et al., 2017). Numerous studies have confirmed that these new subtypes are highly reliable and valid for clinical diagnosis, treatment, and prognostic evaluation (Fan et al., 2021; Dadu et al., 2022). Therefore, identifying biomarkers for the early diagnosis of the PD subtype becomes important (Deng et al., 2022). This is crucial for understanding the pathogenesis of different clinical symptoms exhibited by different subtypes, and the underlying mechanisms that drive the speed of progression. It also holds significant implications for the development of more efficacious, personalized therapeutic interventions.

Fereshtehnejad et al. (2015) found that even with comparable ages of onset and disease duration, the PD-DM subtype manifests more severe symptoms and accelerated clinical progression (Armstrong and Okun, 2020; Erro et al., 2020). Therefore, the early identification of this severe subtype of PD is the focus of our current study. However, our comprehension of the pathogenesis and disease progression mechanisms of these subtypes remains limited to an early-stage detection. Diffusion tensor imaging (DTI) is a robust diagnostic tool that enables the assessment of changes in the microstructural integrity of whole-brain tissue, with a specific capacity to identify the impacts of demyelination and axonal injury on the onset of the disease. The utility of DTI in the early diagnosis and prognostic assessment of PD patients has been demonstrated (Gu et al., 2021; Kamagata et al., 2021; Uhr et al., 2022; Pietracupa et al., 2023). However, most of these studies measured structural changes within small, heterogeneous cohorts of PD patients (e.g., differences in clinical symptoms), leading to inconsistent findings in terms of magnitude and direction (Maetzler et al., 2016; Mole et al., 2016; Wen et al., 2018; Pimer et al., 2023). Only one study has shown that DTI findings in the early de novo phase of PD could be utilized to differentiate clinical subtypes and predict prognosis after 4.5 years (Abbasi et al., 2020), but this study was limited to the basal ganglia region and did not explore structural changes of white matter in the whole brain.

Individual differences in the degeneration of the nigrostriatal dopaminergic pathway and the diverse functional and structural changes within cortico-striatal networks are key contributors to the clinical heterogeneity observed in PD (Chou et al., 2014; Mishra and Dixit, 2021; Chung et al., 2022). Studies have shown that patients with the PD-DM subtype exhibit significantly reduced dopamine transporter (DAT) levels in the putamen and caudate compared to patients with the other two subtypes. Furthermore, DAT levels in the putamen were observed to decline most rapidly over a 2.7-year follow-up period in PD (Fereshtehnejad et al., 2017). Recognizing the potential of DTI as a supplementary tool in diagnosing new PD subtypes, it is important to refine the complementary interpretation of early dopaminergic degeneration based on white matter damage using DTI technology as it is more reliable (Son et al., 2016).

The deposition of α-synuclein and β-amyloid, as well as the neurofibrillary tangles of total tau protein (t-Tau) and phosphorylated tau protein (p-Tau), which affect neurofibrils in PD, are currently topics of significant research interest. α-Synuclein fibrils, known to be involved in the pathogenesis of PD, have been observed to engage in a cell-to-cell propagation through neuronal pathways in the mouse brain following inoculation (Masuda-Suzukake et al., 2013; Zhang S. et al., 2023). Herholz et al. (2016) have reported that in PD patients, microstructural changes observed through DTI are positively correlated with significant reductions in levels of α-synuclein and total tau in the cerebrospinal fluid (CSF). Whether this pattern of injury is also present in the new clinical subtype of PD is a question that warrants further investigation. In addition, patients with the PD-DM subtype exhibit the most rapid cognitive decline and display CSF features similar to Alzheimer's disease, characterized by reduced levels of β-amyloid (Fereshtehnejad et al., 2015). This highlights the crucial role of CSF biomarkers in assessing the prognosis of various subtypes of PD (Abbasi et al., 2018).

In the present study, we hypothesized that white matter damage in different PD subtypes was heterogeneous and correlated with clinical manifestations and disease progression. Changes in DAT activity and neurobiochemical profiles could, to a certain degree, be used to explain the observed white matter damage.

## Materials and methods

### Participants

This study used data extracted from the Parkinson's Progression Markers Initiative (PPMI) database (Marek et al., 2011), encompassing a comprehensive set of clinical, CSF protein, imaging, and genetic markers from PD patients, healthy controls (HCs), and relatives of PD patients. The PD and HC cohorts were matched for age and sex distribution, but they exhibited differences in several key measures, including the Movement Disorder Society-sponsored revision of the Unified Parkinson's Disease Rating Scale (MDS-UPDRS) (Goetz et al., 2008), REM sleep behavior disorder (RBD), Scales for Outcome in Parkinson's Disease-Autonomic (SCOPA-ATU), and Neuropsychological Scale scores. All PD patients were treatment-naïve at baseline. The HCs were free of neurological illnesses. Patients were evaluated with a comprehensive set of clinical tests at baseline and the follow-up visit. We gathered the data of 160 PD patients and 45 HCs aged 45 years or older from PPMI. After excluding 13 participants with missing data and 12 with head motion artifacts, we categorized the remaining 135 PD patients into distinct subtypes based on their baseline scores for composite motor severity and three principal nonmotor domains: RBD, SCOPA-ATU, and cognitive impairment. The PD-MMP subtype included individuals whose composite motor symptom scores and all nonmotor symptom scores were less than the 75th percentile for severity (based on the same PPMI populations). The PD-DM subtype had two classification criteria: individuals with combined motor symptom score and more than one-third of the nonmotor domain scores > the 75th percentile, or with all three nonmotor domain scores > the 75th percentile (regardless of motor severity). The remaining cases were defined as the PD-IM subtype. We also classified the PD patients who continued in the study after 1 year using the criteria for the new clinical subtypes. The study was conducted in accordance with Good Clinical Practice (GCP) regulations and International Conference on Harmonization (ICH) guidelines. The PPMI was a large multicenter study, and ethical approval for the PPMI protocol was independently obtained from the institutional review board at each site. All subjects provided written informed consent in accordance with the Declaration of Helsinki.

### DTI data acquisition

According to the PPMI cohort imaging protocols, 3D-T1 images were acquired using a magnetization-prepared rapid acquisition gradient-echo protocol. The whole-brain diffusion MRI acquisition sequence was done with the following parameters: diffusion gradient direction = 64; TR/TE = 900/88 ms; b-values = 0, 1,000 s/mm^2^; FOV = 230 × 230 mm; in-plane resolution = 2 mm isotropic; 72 slices. The PPMI website shows the details of the acquisition parameters (http://www.ppmi-info.org/study-design/research-documentsand-sops/). Basic quality control assessment of the scans was done at the Neurodegenerative Disease Imaging Center (San Francisco VA Medical Center, San Francisco, CA) and the processed images were uploaded to the PPMI website. Each scan was also visually checked for quality assessment by two physicians with over 10 years of clinical experience but blinded to the status of the subjects.

### DTI image preprocessing

We used FSL version 5.0.2 for image preprocessing, which included head motion eddy correction and gradient direction correction, and the cranial and cervical regions were removed with the BET2 option. Then, a brain mask was generated from the b0 image using the fractional intensity threshold of 0.3 to ensure that only diffusion tensors inside the brain were computed. For individual samples, we made adjustments on a case-by-case basis. Finally, fractional anisotropy (FA) and mean diffusivity (MD) values were computed using the DtiFit tool, in which axial diffusivity (AD) was generated from the L1 vector, and the radial diffusivity (RD) values were obtained from the average of the L2 and L3 vectors.

### Tract-based spatial statistics (TBSS)

Voxel-wise statistical analyses of FA, MD, AD, and RD parameters were performed using TBSS to compare group differences within the white matter skeleton. The mean FA image was created and thinned to form the mean FA skeleton after aligning each FA mapping to the standard space. The threshold of the mean FA skeleton, which included the major white matter pathways but excluded the peripheral bundles and gray matter, representing the centers of all tracts common to the group, was 0.2. Finally, the maximum FA value aligned with each subject was projected onto this skeleton perpendicular to each voxel on the plane of the local skeleton structure. Using the function Tbss_non-FA, the MD, AD, and RD skeletons were generated by applying the aforementioned nonlinear matrix, which was derived from the spatial normalization process of the FA images to the standard space.

### Functional magnetic resonance imaging (fMRI) data acquisition

The MRI data were obtained from the PPMI database. Scans were performed using a standardized protocol, with all participants undergoing resting-state fMRI examination on a 3.0 T MRI scanner (Siemens, TrioTim). The scanner was fitted with an eight-channel phased array head coil, which was activated to minimize head motion. Participants were directed to relax with their eyes closed, remain conscious, maintain stillness, and refrain from focused thinking. Following this preparation, T1-weighted images were acquired using a 3D-T1 gradient-echo sequence [176 axial slices, repetition time (TR) = 2300 ms, echo time (TE) = 3.0 ms, flip angle (FA) = 9°, matrix = 256 × 256, field of view (FOV) = 250 mm × 250 mm, slice thickness = 1 mm, no gap]. Each anatomical run consisted of 176 image volumes covering the entire brain for registration and functional localization. This was followed by the collection of whole-brain gradient-echo sequences (EPI) (TE = 25 ms, TR = 2400 ms, FOV = 240 × 240 mm^2^, slice thickness = 3.3 mm, no gap, matrix size = 476 × 462). A total of 210 time points were recorded for each participant. Throughout the fMRI acquisition, participants were instructed to relax, keep their eyes open to avoid falling asleep, and their status was confirmed immediately after the experiment ended. Earplugs and pads were used to minimize scanner noise and head movement.

### fMRI data preprocessing

The resting-state network data were analyzed with graph theoretical network analysis toolbox (GRETNA; https://www.nitrc.org/). All software programs were run on a Statistical Parametric Mapping platform (SPM12; http://www.fil.ion.ucl.ac.uk/spm) and GroupICATv4.0b software (http://www.restfmri.net). All DICOM files were converted into Neuroimaging Informatics Technology Initiative (NIfTI) files. For each participant, the initial five time points of the fMRI series were discarded to allow for signal equilibration and participant acclimatization to the scanning environment and noise. Next, the following preprocessing steps were performed on the functional images: slice timing, motion correction, spatial standardization to the Montreal Neurological Institute (MNI) EPI template (resampling voxel size = 3 × 3 × 3 mm^3^) in SPM12, linear trend removal and temporal bandpass filtering (0.01–0.08 Hz) on the time series of each voxel. Then, covariates such as white matter signals, CSF signals, and motion signals were regressed. Further analysis included only subjects with < 1.5 mm of maximum displacement in the x-, y-, or z-plane and < 1.5° of angular rotation on each axis.

### Independent component analysis (ICA)

The single scans of participants were merged into a 4D file in SPM12. ICA was performed on the merged file in the GIFT toolbox (http://mialab.mrn.org/software/gift/, RRID:SCR_001953). The number of independent components was set to 30. The criterion for screening was that the peak point of the component was located in the gray matter with a frequency < 0.1. Simultaneously, we incorporated the resting default mode network (DMN) template for an automatic recognition of the component.

### Single-photon emission computed tomography (SPECT)

I-123 FP-CIT SPECT images from the respective institutions were managed by the core imaging lab of PPMI. The core lab performed quality control to maintain reliable data quality. I-123 FP-CIT SPECT images were taken 4 ± 0.5 h after I-123 FP-CIT injection (111-185 MBq). Images were reconstructed iteratively with no filtering. PMOD software (PMOD Technologies, Zurich, Switzerland) was used for the calculation of specific binding ratios [SBRs, (target region/reference region) – 1] of bilateral caudate and putamen with the occipital cortex as the reference.

SPECT with the DAT tracer 123I-ioflupane was obtained from 176 PPMI participants at baseline (Marek et al., 2011). The striatal binding ratio (SBR) was calculated for the caudate putamen (left, right, and mean).

### Cerebrospinal fluid (CSF) biomarker assessments

CSF was collected at each study site as described in the PPMI biologics manual (http://www.ppmi-info.org/). β-Amyloid, t-Tau, and p-Tau181 concentrations in the CSF were analyzed at the University of Pennsylvania as previously described (Kang, 2013). The α-synuclein concentration in the CSF was analyzed at Covance (Princeton, NJ) using a commercially available enzyme-linked immunosorbent assay kit (Mollenhauer et al., 2013).

### Statistical analysis

#### Demographics

Demographics and clinical features were assessed with Statistical Package for Social Sciences (SPSS version 26) software. Independent t-tests and analysis of variance (ANOVA) were used to evaluate the differences in continuous variables that were normally distributed, and the Friedman test was applied to continuous data that did not conform to a normal distribution. *Post hoc* Bonferroni multiple-comparison tests and the Mann-Whitney U test were employed to explore significant group effects. Categorical variables were assessed using the chi-square test (x^2^).

#### DTI analysis

All voxel-wise comparisons were performed using a permutation-based inference tool for nonparametric thresholding (FSL's “randomize”) (Smith and Nichols, 2009; Winkler et al., 2014). Group effects were investigated for each of the four imaging metrics (FA, MD, AD, and RD). Comparisons between groups, including PD-DM subtype, PD-IM subtype, and PD-MMP subtype with HCs, were done separately at baseline. The randomize function in FSL was employed to perform 5000 permutations, and the outcomes were corrected for threshold-free cluster enhancement (TFCE) and for multiple comparisons using family-wise error correction (FWE). Statistical significance was set at *p* < 0.05, taking into account the effects of age, sex, and family history. In order to strengthen the statistical rigor of the TBSS analysis, the threshold for statistical significance of FA values was set to *p* < 0.033. Regions exhibiting significant differences between groups were identified by overlaying the statistical results onto the Johns Hopkins University DTI white matter atlas within FSL and the DMN. For these regions of interest with significant differences, we utilized the seed points for probabilistic fiber tracking. Further details of the methodology can be found in the Supplementary material.

To analyze the overall disease progression of PD patients, we employed the previously published global composite outcome (GCO) as a single numerical indicator for prognosis. The GCO equally weighted nonmotor symptoms (MDS-UPDRS I), motor symptoms (MDS-UPDRS II), motor signs (MDS-UPDRS III), overall activities of daily living (Schwab and England ADL), and Montreal Cognitive Assessment (MOCA) scores, which were standardized by averaging the z scores of each component (Fereshtehnejad et al., 2015, 2017). To determine changes at 1 year, we used the mean ± SD from baseline as a reference. The total GCO score for each subtype of PD patients was derived by averaging all the component z scores, with higher GCO scores indicating poorer functional performance.

To determine the clinical significance of the DTI findings, the significant clusters identified in the above voxel-based analyses were saved as binary masks. The global mean values of the corresponding voxel-based masks were then extracted for each participant, and their correlations with motor symptom scale scores, cognitive function scale scores, and CSF protein levels were determined through multiple linear regression (MLR), with p < 0.05 (Bonferroni corrected) indicating statistical significance, adjusted for age, relationship of PD family, and education (years). Additionally, the covariate included disease duration (months), levodopa equivalent daily dose (LEDD), and scanning intervals (years) for follow-up data.

In addition, the MLR was applied to investigate the effect of reduced DAT uptake in the caudate putamen region on the aforementioned white matter damage and on clinical symptoms at baseline and after follow-up. Age, family history, and disease duration were used as baseline relevant variables, but LEDD was additionally added for follow-up data.

## Results

**Figure 1:**
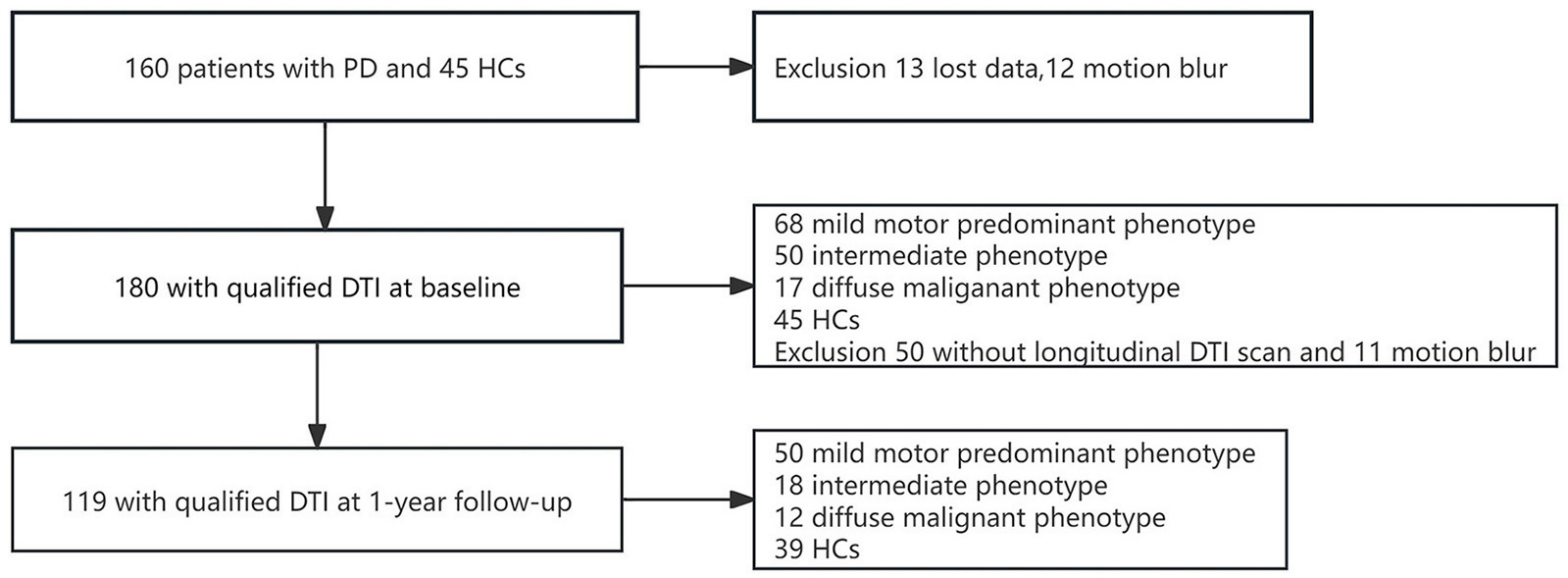
Flowchart showing the procedure for filtering DTI images of PD patients and HCs at baseline and 1-year follow-up.

The baseline analyses were performed for 180 participants (45 HCs, 68 PD-MMP patients, 50 PD-IM patients, and 17 PD-DM patients) who completed the clinical assessment and had high-quality baseline DTI scans. After 1 year, 119 participants (39 HCs, 50 PD-MMP patients, 18 PD-IM patients, and 12 PD-DM patients) completed the follow-up clinical assessment and were rescanned successfully (Figure 1). The subtypes of PD patients who remained in the study did not change at 1-year follow-up.

### Demographics and clinical features

Table 1 summarizes the demographic and clinical features of the groups. At baseline, the sex distribution and age did not significantly differ between patients with PD and HCs. Among patients with the PD-DM subtype, the proportion of males was the highest among all the groups at the 1-year follow-up. Therefore, we performed a series of sensitivity analyses and demonstrated that the results were robust (Supplementary Table 1). In addition, differences in family history were detected between the HCs and the other three subtypes. Educational time (years), disease duration (months), and LEDD did not show significant difference among the groups. At baseline and after 1-year follow-up, there were significant differences between groups in the MDS-UPDRS subscales, Postural Instability and Gait Disorder (PIGD) scores, Hoehn and Yahr stage, RBD, and SCOPA-ATU. At baseline, the patients with PD-DM subtype showed significant differences compared to HCs and patients with the PD-MMP subtype in the Benton Judgment of Line Orientation, recognition discrimination in the Hopkins Verbal Learning Test (HVLT), semantic verbal fluency, Symbol Digit Test, and MOCA. After 1-year follow-up, the patients with the PD-DM subtype demonstrated significant differences relative to HCs in the Benton Judgment of Line Orientation, HVLT—total recall, HVLT—delay recall, HVLT—recognition discrimination, and semantic verbal fluency. Significant differences were also found among all groups in the Symbol Digit Test and MOCA after 1-year follow-up. At baseline, both the levels of α-synuclein and β-amyloid in the CSF in the patients with the PD-DM subtype were significantly lower compared to HCs. After 1-year follow-up, the levels of α-synuclein in the CSF in the patients with the PD-DM subtype continued to be significantly lower than those in HCs.

**Table 1:** Demographic features of the study cohort.

**Characteristic**	**HCs**		**PD-MMP**		**PD-IM**		**PD-DM**		***P*** **-value**	
	**Baseline (*n* = 45)**	**Follow-up (*n* = 39)**	**Baseline (*n* = 68)**	**Follow-up (*n* = 50)**	**Baseline (*n* = 50)**	**Follow-up (*n* = 18)**	**Baseline (*n* = 17)**	**Follow-up (*n* = 12)**	**Baseline**	**Follow-up**
Female %^a^	42.20%	41.10%	36.76%	36.00%	48.00%	34.00%	22.22%	0.00%	0.307	0.052
Age (years)	61.4 (8.2)	63.3 (8.1)	60.5 (9.6)	61.4 (9.4)	61.4 (9.3)	61.9 (8.9)	67.3 (8.8)	67.0 (9.3)	0.064	0.231
Educational time (years)	15.7 (3.0)	15.7 (2.7)	15.7 (2.9)	15.3 (2.8)	15.0 (2.8)	14.9 (2.4)	16.6 (3.3)	16.0 (2.4)	0.377	0.529
Relationship in PD	0.04 (0.2)	0.05 (0.2)	0.5 (0.9)	0.3 (0.7)	0.12 (0.3)	0.09 (0.2)	0.5 (0.5)	0.5 (0.1)	< 0.001 (DM vs. HCs)	0.006 (DM vs. HCs, MMP vs. HCs)
Duration of disease (months)^b^	/	/	7.3 (7.3)	7.4 (7.4)	7.2 (6.8)	7.1 (5.1)	9.8 (8.5)	10.3 (8.6)	0.073	0.481
LEDD (mg/day)^b^	/	/	/	262.2 (151.2)	/	262.3 (152.4)	/	306.6 (175.5)	/	0.578
**Motor symptoms and signs**										
MDS-UPDRS II	0.2 (0.5)	0.4 (1.3)	3.2 (2.8)	5.6 (3.4)	5.8 (3.8)	8.3 (4.2)	11.5 (4.9)	11.7 (3.7)	< 0.001 (All comparisons)	< 0.001 (All comparisons)
MDS-UPDRS III	0.7 (1.1)	1.0 (2.0)	17.6 (7.4)	18.6 (9.6)	19.8 (8.0)	24.4 (11.1)	31.5 (7.4)	31.8 (7.9)	< 0.001 (All comparisons)	< 0.001 (All comparisons)
MDS-UPDRS-total	3.6 (3.1)	2.6 (2.2)	24.9 (9.6)	29.2 (13.1)	31.5 (11.7)	38.3 (17.1)	53 (10.1)	54.4 (13.3)	< 0.001 (All comparisons)	< 0.001 (All comparisons)
PIGD score	0 (0)	0.01 (0.04)	0.14 (0.17)	0.2 (0.1)	0.2 (0.1)	0.3 (0.2)	0.6 (0.3)	0.6 (0.3)	< 0.001 (All comparisons)	< 0.001 (All comparisons)
Hoehn and Yahr stage	0 (0)	0.08 (0.3)	1.5(0.5)	1.6 (0.5)	1.6 (0.4)	2.0 (0.6)	2.0(0.4)	1.9 (0.2)	0.022 (All comparisons)	< 0.001 (All comparisons)
S&E-ADL^b^	/	/	95.9 (4.3)	93.6 (5.1)	93.9 (6.3)	89.1 (6.9)	89.7 (6.2)	89.23 (6.1)	0.134	0.366
**Nonmotor symptoms and signs**										
MDS-UPDRS I	0.69 (1.58)	2.6 (2.2)	0.9 (1.3)	3.8 (2.8)	5.8 (3.6)	5.8 (3.5)	2.6 (3.3)	8.9 (3.9)	< 0.001 (All comparisons)	< 0.001 (All comparisons)
RBD	3.7 (1.9)	2.6 (1.9)	2.8 (1.4)	2.6 (1.9)	5.1 (2.4)	4.7 (3.2)	7.4 (2.8)	7.0 (3.4)	< 0.001 (All comparisons)	< 0.001 (All comparisons)
Benton Judgment of Line Orientation	12.2 (2.6)	11.5 (3.6)	12.6 (2.7)	11.0 (3.1)	11.6 (2.9)	10.9 (2.6)	10.2 (3.7)	9.2 (2.3)	0.002 (DM vs. HCs, DM vs. MMP)	0.001 (DM vs. HCs)
SCOPA-ATU	10.8 (9.0)	6.2 (4.2)	7.2 (4.4)	6.2 (4.2)	10.8 (6.6)	11.0 (5.2)	18.3 (8.2)	18.1 (5.8)	< 0.001 (All comparisons)	< 0.001 (All comparisons)
**Cognition**										
HVLT-total recall (T-score)	50.1 (9.4)	51.8 (8.2)	48.2 (10.6)	47.8 (9.4)	46.3 (12.8)	44.6 (13.3)	43.1 (11.6)	40.5 (13.9)	0.114	0.005 (DM vs. HC)
HVLT-delayed recall (T-score)	49.7 (8.3)	50.9 (8.0)	47.6 (10.7)	48.0 (10.2)	45.5 (12.7)	44.4 (12.8)	43.2 (11.4)	39.5 (12.5)	0.108	0.004 (DM vs. HC)
HVLT-rentention (T-score)	50.6 (7.6)	49.8 (9.6)	48.9 (10.9)	49.0 (9.9)	46.1 (11.2)	45.2 (12.5)	46.6 (10.2)	44.6 (13.4)	0.136	0.183
HVLT-recognition discriminition (T-score)	48.9 (10.2)	51.7 (10.6)	47.7 (10.0)	45.8 (11.5)	45.4 (11.1)	45.7 (12.4)	39.1 (14.4)	41.3 (16.5)	0.009 (DM vs. HCs, DM vs. MMP)	0.005 (DM vs. HCs)
Letter number sequencing score	11.69 (2.4)	11.6 (2.3)	12.0 (2.3)	11.6 (2.4)	11.1 (2.7)	11.3 (2.5)	11.3 (2.4)	11.2 (3.1)	0.202	0.911
Semantic verbal fluency (T-score)	54.1 (9.6)	53.3 (8.4)	52.7 (9.7)	53 (9.3)	51.3 (8.0)	55.0 (12.2)	50.2 (2.4)	49.0 (8.3)	< 0.001 (DM vs. HCs, DM vs. MMP)	0.029 (DM vs. HCs)
Symbol digit test (T-score)	49.6 (8.7)	51.1 (10.8)	46.6 (7.7)	42.7 (9.8)	41.6 (11.0)	39.9 (9.1)	41.2 (9.3)	36.3(11.1)	< 0.001 (DM vs. HCs, DM vs. MMP)	< 0.001 (All comparisons)
MOCA	28.2 (1.1)	27.4 (1.9)	28 (1.9)	27.4 (2.3)	27.3 (2.3)	26.3 (3.4)	26.2 (2.0)	24.6(3.9)	< 0.001 (DM vs. HCs, DM vs. MMP)	< 0.001 (All comparisons)
**CSF**										
β-Amyloid	978.2 (421.7)	1027.7 (410.0)	840.0 (362.7)	871.5 (390.0)	856.7 (355.5)	892.4 (377.3)	840.8 (360.9)	776.9 (283.4)	0.017 (DM vs. HCs)	0.135
α-Synuclein	1,548.8 (609.0)	1,803.8 (692.0)	1,407.2 (600.5)	1,461.8 (637.8)	1,453.0 (555.6)	1,363.5 (454.6)	1,248.0 (500.3)	1,072.0 (554.0)	0.012 (DM vs. HCs)	0.004 (DM vs. HCs)
t-Tau	177.0 (66.6)	194.6 (70.3)	162.3 (49.4)	166.2 (52.3)	169.5 (55.5)	167.7 (46.2)	153.6 (41.7)	142.3 (44.6)	0.593	0.142
p-Tau	16.4 (6.4)	17.4 (6.9)	14.2 (4.7)	14.4 (5.0)	14.3 (4.9)	14.4 (4.2)	13.1 (3.6)	13.5 (4.0)	0.155	0.056

Means and standard deviations (SD) are reported; P-value is derived from the analysis of variance (ANOVA) test (F test) for four groups, Bonferroni *post hoc* analysis shows difference in groups; ^a^Analysis using chi-square or Fisher's exact test; ^b^Comparison between three subtypes. In longitudinal study, all scales were assessed during the off-medication phase—that is, after the patient had refrained from regular anti-PD medication for 12 h overnight. LEDD, Levodopa equivalent daily dose; MDS-UPDRS, Movement Disorder Society-sponsored revision of the Unified Parkinson's Disease Rating Scale; PIGD, Postural Instability and Gait Disorder; S&E-ADL, Schwab and England activities of Daily Living Scale; RBD, REM sleep behavior disorder; SCOPA-ATU, Scales for Outcome in Parkinson's Disease-Autonomic; HVLT, Hopkins verbal learning test; MOCA, Montreal Cognitive Assessment; MMP, mild motor predominant; IM, intermediate; DM, diffuse malignant.

### Baseline diffusion tensor imaging parameters

**Figure 2:**
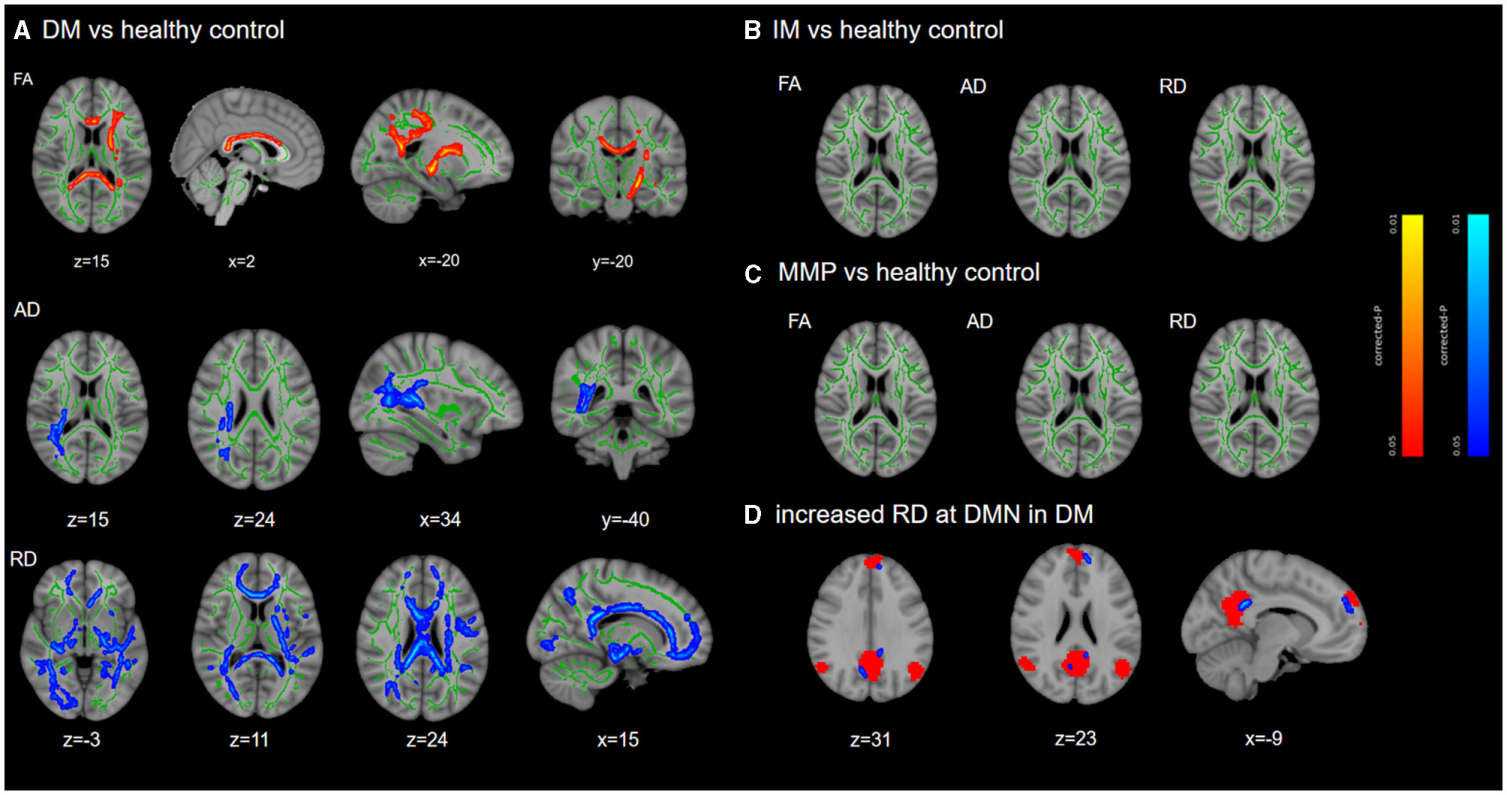
TBSS differences at baseline. TBSS analyzed the differences in white matter damage between each subtype and HCs. Results are overlaid on the Montreal Neurological Institute standard brain. The FA skeleton is shown in green, while red/blue indicates significant decreased/increased region and displayed at *p* < 0.05 (FWE corrected) in **(A)**. There were no significant differences among PD-MMP subtype and PD-IM subtype compared to HCs **(B, C)**. The red mask in **(D)** indicates the DMN network. DM, diffuse malignant; IM, intermediate; MMP, mild motor predominant.

At baseline, compared with HCs, patients with the PD-DM subtype had significantly lower FA values in the commissural fibers (corpus callosum), projection fibers (left superior and posterior corona radiata, left corticospinal tract, left internal capsule), association fibers (left inferior longitudinal fasciculus, left inferior frontal-occipital fasciculus). RD, including in the extensive white matter areas of the commissural fibers, association fibers, and projection fibers, was significantly higher. In addition, significantly higher AD values were observed in the association fibers (right superior and inferior longitudinal fasciculus, right inferior frontal-occipital fasciculus) and projection fibers (right corticospinal fasciculus, right posterior limb of the internal capsule, and right superior and posterior corona radiata), as well as corpus callosum (accepted by Probtrackx) (Figure 2A, Supplementary Figure 1). There were no significant differences in these areas among the PD-MMP and PD-IM patients compared to HCs (Figures 2B, C). MD values were not significantly different in any of the group comparisons. We recognized that RD in patients with the PD-DM subtype was elevated in the medial prefrontal cortex (mPFC) and posterior cingulate cortex (PCC) adjacent to white matter by DMN (Figure 2D).

**Figure 3:**
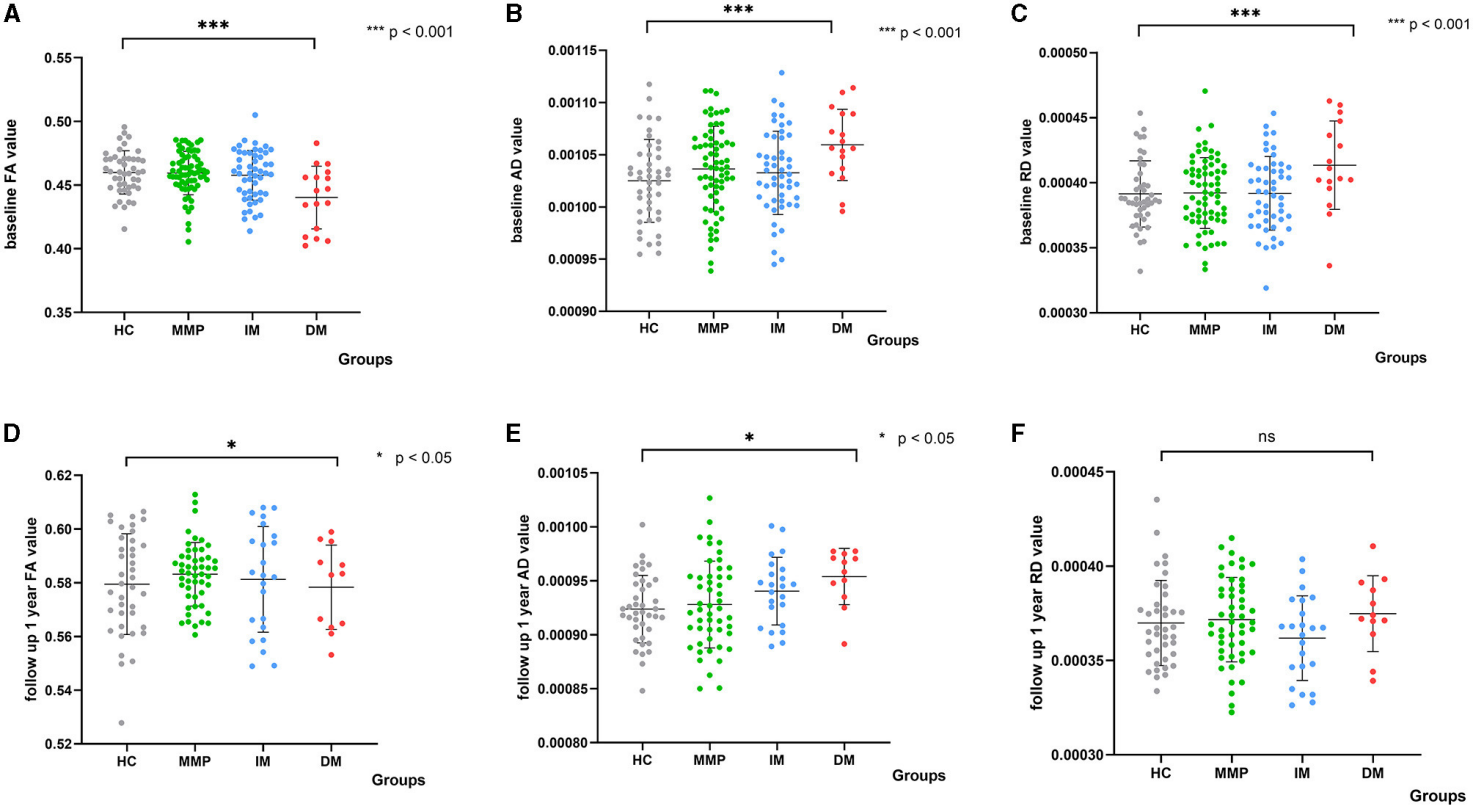
Scatter plots depict diffusion parameters for PD subtypes and HCs at baseline and after 1 year. The white matter damage region is highlighted by comparing PD-DM versus HCs at baseline. Analysis of covariance (ANCOVA) differentiated diffusion parameter values between each subtype and HCs. **(A–C)** Show differences at baseline; **(D–F)** Show differences at 1-year follow-up. Analysis corrected for age, sex, and relationship of PD family. FA, fractional anisotropy; AD, axial diffusivity; RD, radial diffusivity; DM, diffuse malignant; IM, intermediate; MMP, mild motor predominant. ^***^indicated a significant p-value of less than 0.001, and ^*^indicated a significant p-value of less than 0.05.

To ensure the stability and reliability of our experimental outcomes, we employed the leave-one-out (LOO) cross-validation method. This approach maximizes the utilization of data for validation, thereby enhancing the accuracy of our classifier. The classification results we obtained are consistent and deterministic. We further transformed the aforementioned regions into masks and aligned them to the baseline data as well as follow-up data of all PD patients and HCs. The results are presented in Figure 3.

### Baseline correlation of diffusion parameter values with clinical symptoms

**Figure 4:**
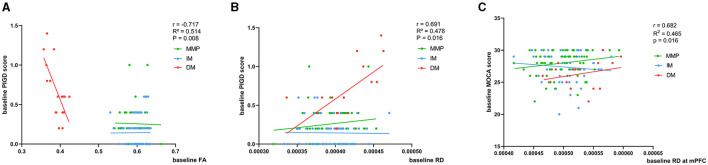
**(A–C)** Correlation of baseline FA and RD values with clinical scores. Age, relationship of PD family, and educational time (years) are the other regression variables. r, R2, and p demonstrate the correlation coefficient, determination coefficient, and significance, respectively. PIGD, Postural Instability and Gait Disorder score; MOCA, Montreal Cognitive Assessment; FA, fractional anisotropy; RD, radial diffusivity; DM, diffuse malignant; IM, intermediate; MMP, mild motor predominant. The green, blue, and red lines respectively represented MMP, IM, and DM.

At baseline, using MLR analysis with adjustment for confounders to explore the association of diffusion parameters with motor and nonmotor symptom severity in PD patients, we found a linear relationship between FA and RD values and PIGD scores in patients with the PD-DM subtype (r = −0.717, *p* = 0.008, r = 0.691, *p* = 0.016) (Figures 4A, B, Table 2A). Abnormal RD values in the DMN, particularly in the white matter adjacent to mPFC, were related to the Montreal Cognitive Assessment (MOCA) scores (r = 0.682, *p* = 0.016) (Figure 4C, Table 2B). AD values showed no correlation with any clinical scale scores at baseline.

**Table 2:** Baseline correlation of diffusion parameter values with motor and nonmotor symptoms.

**A**	**Baseline PIGD score**	**Baseline FA**			**Baseline RD**		
		**β (95% CI)**	**r**	***p***	**β (95% CI)**	**r**	***p***
	MMP	−0.29 (−1.80; 2.40)	0.226	0.778	−298.03 (−1,964.65; 1,368.52)	0.228	0.722
	IM	−0.02 (−0.04; −0.01)	0.407	0.935	51.66 (−1,040.40; 1,144.01)	0.407	0.925
	DM	−13.04 (−21.90; −4.18)	−0.717	0.008	8,476.682 (1,716.77; 15,237.72)	0.691	0.016
**B**	**MOCA score**	**Baseline RD at mPFC**		
		**β (95% CI)**	**r**	***p***
	MMP	12,631.45 (−800.23; 26,063.51)	0.352	0.065
	IM	608.53 (−21,673.23; 22,890.23)	−0.272	0.965
	DM	8,476.682 (1,716.77; 15,237.72)	0.682	0.016

Analyses were controlled for age, relationship of PD family, education (years). 95% confidence interval, correlation coefficients (rs) and p values (Bonferroni corrected) are presented. FA, fractional anisotropy; RD, radial diffusivity; PIGD, Postural Instability and Gait Disorder; MOCA, Montreal Cognitive Assessment; DM, diffuse malignant; IM, intermediate; MMP, mild motor predominant.

### Longitudinal correlation of diffusion parameter values with clinical symptoms

**Figure 5:**
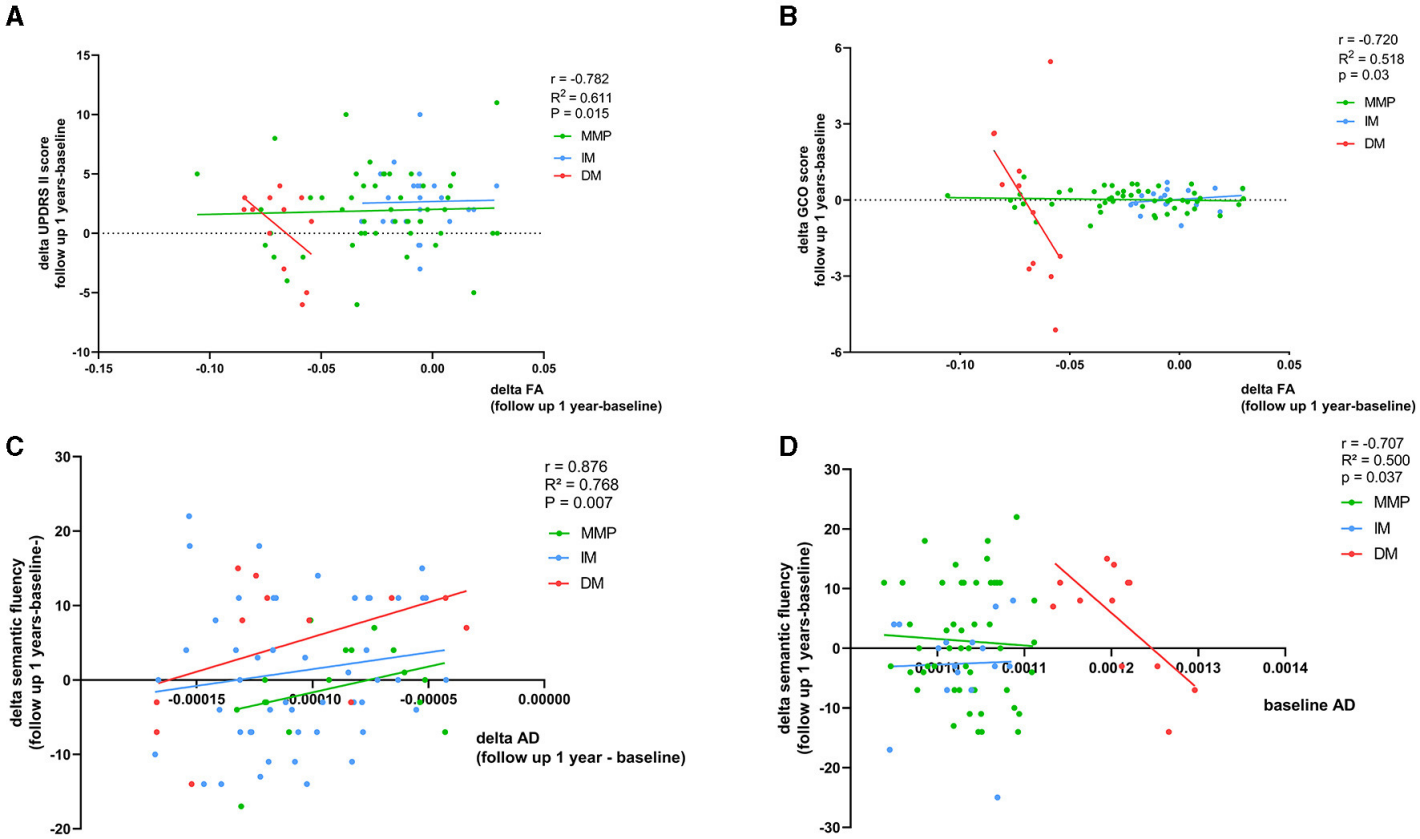
**(A–D)** Correspondence of delta FA and AD values and baseline AD value with delta clinical scales scores. Age, relationship of PD family, educational time (years), disease duration (months), LEDD (mg/day), and interval between visits (years) as other regression variables. r, R2, and p demonstrate the correlation coefficient, determination coefficient, and significance, respectively, in PD-DM subtype. GCO, global composite outcome. The green, blue, and red lines respectively represented MMP, IM, and DM.

We found that a faster decrease in FA was related to a faster increase in the motor subscale score (MDS-UPDRS II) in patients with the PD-DM subtype (r = −0.782, *p* = 0.015) (Figure 5A, Table 3A). There were no significant correlations among AD, RD, and motor function at the follow-up visits in patients with PD. We calculated the GCO at baseline and after 1-year follow-up. In the longitudinal study of the PD-DM subtype population, an increase in the GCO score was associated with a decrease in the FA value (r = −0.720, *p* = 0.03) (Figure 5B, Table 3A). However, none of the remaining parameter values correlated with the GCO score at 1-year follow-up. For nonmotor symptoms, the area of the right temporal-parietal fiber intersection, which exhibited a rapid decline in AD, was correlated with a worsening of semantic verbal fluency in patients with the PD-DM subtype (r = 0.876, *p* = 0.007) (Figure 5C, Table 3B). There were no significant correlations among FA, RD, and nonmotor functions.

**Table 3:** Longitudinal correlation of diffusion parameter values with motor and nonmotor symptoms.

**A**	**Delta FA**	**Delta MDS-UPDRS II score**			**Delta GCO**		
		**β (95% CI)**	**r**	***p***	**β (95% CI)**	**r**	***p***
	MMP	4.428 (−39.98; 43.83)	0.049	0.797	−0.773 (−5.05; 3.50)	0.718	0.133
	IM	−12.38 (−97.72; 72.05)	0.535	0.764	−0.425 (−27.26; 26.41)	0.973	0.266
	DM	−339.27 (−588.80; 39.74)	−0.782	0.015	−285.44 (−534.60; −36.27)	−0.72	0.03
**B**	**Delta AD**	**Delta semantic verbal fluency score**		
		**β (95% CI)**	**r**	***p***
	MMP	−50,10.82 (−32,657.81; 22,636.52)	0.062	0.717
	IM	−26,956.98 (−288,592.41; 234,678.44)	0.287	0.827
	DM	150,189.05 (49,986; 250,391)	0.876	0.007

The correlation between change of diffusion parameters (FA, AD) and clinical symptom (MDS-UPDRS II, GCO, semantic verbal fluency score) at 1-year follow-up are displayed in this table. Delta value was calculated by 1-year follow-up parameters or clinical scores—baseline parameters or clinical scores. Analyses were controlled for age, relationship of PD family, education (months), disease duration (years), LEDD (mg/day) and interval between visits (years). 95% confidence interval, correlation coefficients (rs), and p values (Bonferroni corrected) are presented.

### Correlation of baseline diffusion parameters with delta clinical scales scores at 1-year follow-up

In patients with the PD-DM subtype, higher AD values at baseline predicted a more significant deterioration in the semantic verbal fluency test score after 1 year (r = −0.707 *p* = 0.037) (Figure 5D, Table 4). No correlation was found between RD and FA values at baseline and the changes in clinical scale scores after 1 year in patients with the PD-DM subtype.

**Table 4:** Correlation of baseline AD values with the change of semantic verbal fluency score.

**Baseline AD**	**Delta semantic verbal fluency score**		
	**β (95% CI)**	**r**	***p***
MMP	−9,808.83 (−75,413.81; 55,769.52)	−0.125	0.765
IM	−52,155.56 (−117,332.41; 221,642.44)	0.294	0.520
DM	−116,535.05 (−223,827.42; 9,242.15)	−0.707	0.037

The correlation between baseline AD value and the change of semantic verbal fluency score at 1-year follow-up are displayed in this table. Delta value was calculated by 1-year follow-up clinical scores – baseline clinical scores. Analyses were controlled for age, relationship of PD family, education (years), disease duration (months), LEDD (mg/day) and interval between visits (years). 95% confidence interval, correlation coefficients (rs), and p values (Bonferroni corrected) are presented.

### Correlation of diffusion parameters with CSF-related protein concentrations

**Figure 6:**
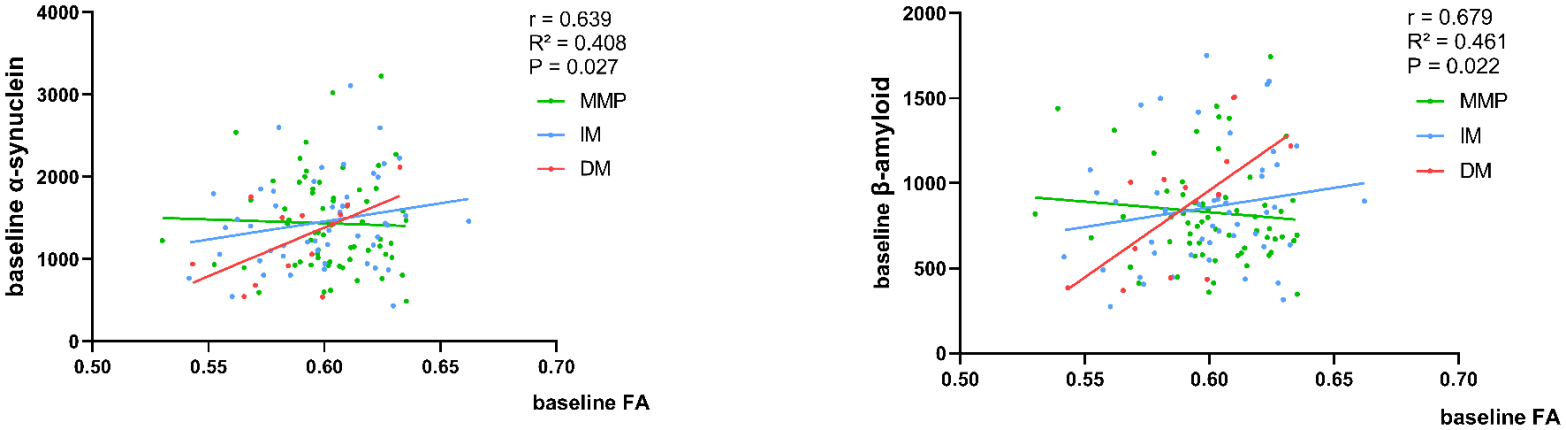
Correlation of baseline FA values with CSF α-synuclein and β-amyloid concentration. Analysis corrected for age. The green, blue, and red lines respectively represented MMP, IM, and DM.

At baseline, in patients with the PD-DM subtype, a rapid decline in FA values was associated with a diminished concentration of α-synuclein and β-amyloid in the CSF (r = 0.639, *p* = 0.027; r = 0.679, *p* = 0.022) (Figure 6). None of the remaining diffusion parameter values correlated with CSF-related protein concentrations.

### Correlation of reduced DAT uptake with clinical scales scores at baseline and follow-up

**Figure 7:**
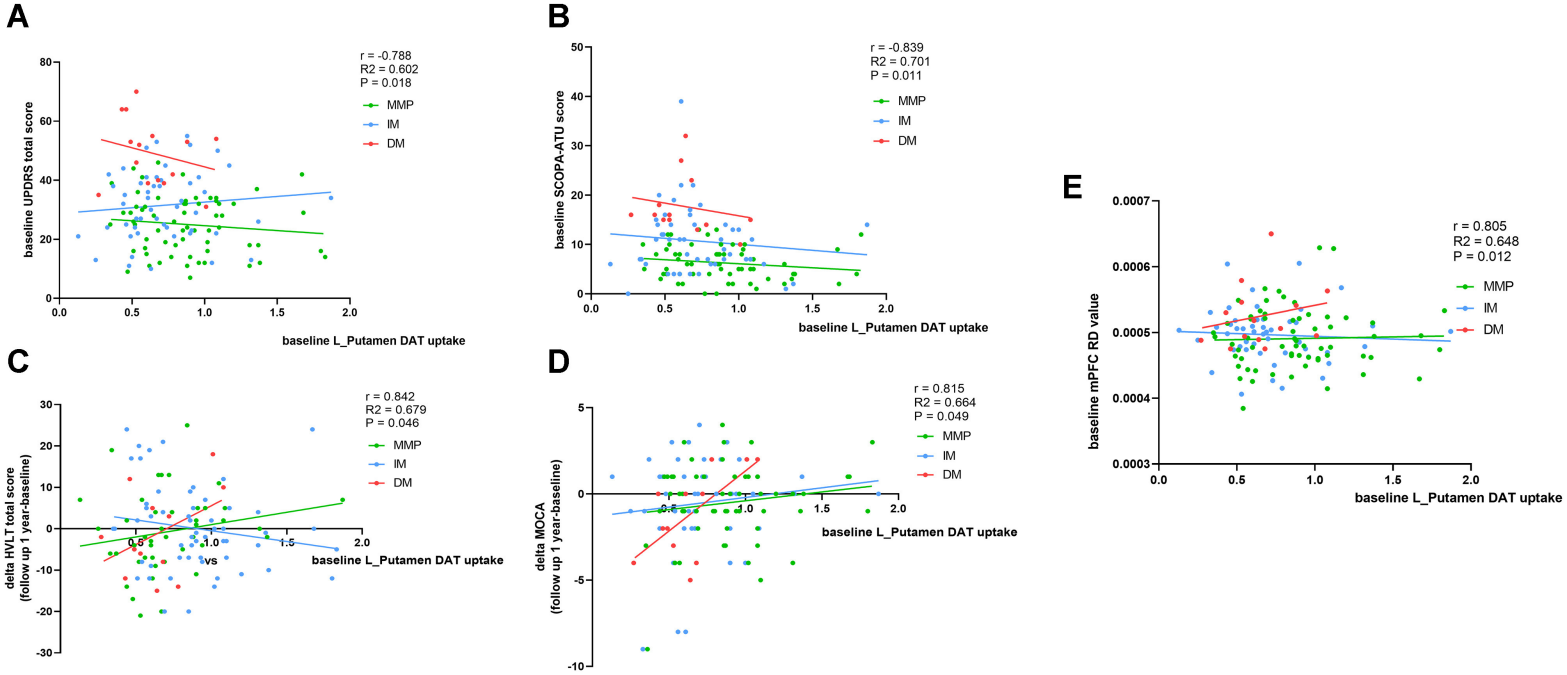
Correspondence of DAT uptakes with clinical scores and mPFC RD value. Analysis showed correlation of L_putamen DAT uptakes with clinical scores and mPFC RD value at baseline and 1 year later. **(A, B, E)** Corrected for age, relationship of PD family, and disease duration (months). **(C, D)** Additionally add LEDD as relevant variables. r, R^2^, and p demonstrate the correlation coefficient, determination coefficient, and significance, respectively, in patients with the PD-DM subtype. SCOPA-ATU, Scales for Outcome in Parkinson' s Disease -Autonomic; MOCA, Montreal Cognitive Assessment; mPFC, medial prefrontal cortex. The green, blue, and red lines respectively represented MMP, IM, and DM.

We evaluated DAT uptake in the caudate putamen (left, right, and mean) for each subtype at baseline. Patients with the PD-DM subtypes exhibited lower DAT uptake in the putamen and caudate (left, right and mean) than the other two subtypes patients, and L-Putamen showed lower DAT uptake than the other regions (Table 5). MLR analyses revealed that reduced DAT uptake in the L-Putamen at baseline was associated with worsening of the MDS-UPDRS total score, SCOPA-ATU score (r = −0.788, *p* = 0.018; r = −0.839, *p* = 0.011), and with changes in the MOCA and HVLT—total scores at 1-year follow-up (r = 0.842, *p* = 0.046; r = 0.839, *p* = 0.049) (Figures 7A–D, Table 6). We subsequently found a correlation between reduced L_putamen DAT uptake and RD in the white matter adjacent to mPFC at baseline in patients with the PD-DM subtype (r = 0.805, *p* = 0.012) (Figure 7E, Table 6).

**Table 5:** Comparison of DAT uptakes between three subtypes.

**Characteristic**	**MMP**	**IM**	**DM**	***P*-value**
R_Caudate	2.071 (0.558)	1.874 (0.478)	1.560 (0.463)	0.004 (DM vs. MMP)
L_Caudate	2.089 (0.585)	1.806 (0.533)	1.473 (0.496)	< 0.001 (DM vs. MMP, IM vs. MMP)
Mean_Caudate	2.080 (0.509)	1.840 (0.453)	1.516 (0.468)	< 0.001 (DM vs. MMP, IM vs. MMP)
R_Putamen	0.891 (0.330)	0.774 (0.276)	0.679 (0.214)	0.025 (DM vs. MMP, IM vs. MMP)
L_Putamen	0.885 (0.342)	0.721 (0.311)	0.644 (0.221)	0.005 (DM vs. MMP, IM vs. MMP)
Mean_Putamen	0.888 (0.259)	0.748 (0.239)	0.656 (0.164)	< 0.001 (DM vs. MMP, IM vs. MMP)

Means and standard deviations of DAT uptakes in left, right, and mean caudate putamen are reported in this table. P values indicate the significance of the ANOVA (F) for the three subtypes, and the results of Bonferroni *post hoc* analyses are shown in brackets.

**Table 6:** Correlation of baseline L_Putamen uptakes with clinical scales and DTI parameter.

**Phenotype**	**MMP**	**IM**	**DM**
**Correlation r(p)**			
L_Putamen with MDS-UPDRS total score^a^	−0.293 (0.145)	−0.233 (0.940)	−0.788 (0.018)
L_Putamen with SCOPA-ATU score^a^	−0.234 (0.358)	−0.153 (0.517)	−0.839 (0.011)
L_Putamen with delta HVLT-total score^b^	−0.345 (0.288)	0.325 (0.370)	0.842 (0.046)
L_Putamen with delta MOCA score^b^	0.636 (0.258)	0.371 (0.398)	0.815 (0.049)
L_Putamen with mPFC_RD value^a^	0.302 (0.600)	0.427 (0.909)	0.805 (0.012)

MLR analysis was applied in this table. ^a^Age, relationship of PD family and disease duration as relevant variables. ^b^Age, relationship of PD family, disease duration and LEDD as relevant variables. Delta shows by 1-year follow-up DAT uptakes - baseline. Coefficients (rs) and P values (Bonferroni corrected) are presented in brackets. DAT, dopamine transporter; mPFC, medial prefrontal cortex.

## Discussion

Our study yielded four major findings. Firstly, at baseline, a comparative analysis revealed that patients with the PD-DM subtype exhibited significantly reduced FA and increased AD and RD in white matter tracts. These alterations were found to correlate with the severity of clinical symptoms and the levels of α-synuclein and β-amyloid in the CSF. In contrast, patients with the PD-MMP subtype and PD-IM subtype did not exhibit significant differences. Secondly, our follow-up analysis revealed that decreased FA values were associated with worsening MDS-UPDRS II scores and GCO scores in patients with the PD-DM subtype and decreased AD values correlated with a deterioration in semantic verbal fluency scores within this patient cohort. The study findings indicated that changes in DTI parameters partially mirrored the pathological progression closely associated with PD-DM patients' clinical symptoms. These results initially revealed distinct pathological trajectories in patients with the PD-DM subtype. Thirdly, this study identified that AD value as a significant predictor of decline in semantic verbal fluency in the early stages of PD-DM subtype after 1-year period. Lastly, diminished DAT uptake in the left putamen was associated with subcortical white matter damage of the mPFC, which was related to motor symptoms and SCOPA-ATU score at baseline. Furthermore, this uptake was a prognostic indicator of memory loss and cognitive decline over the subsequent year.

Among the three subtypes, the PD-DM subtype is widely recognized to be associated with more severe motor and nonmotor symptoms. Bradykinesia, resting tremor, and rigidity are the hallmark motor manifestations of PD (Ye et al., 2023). Our findings indicated a reduction in FA predominantly in the corpus callosum (CC), the left internal capsule, the left posterior corona radiata (PCR), and the left corticospinal tract (CST). This reduction exhibited a linear correlation with PIGD scores, an association that was exclusive to patients categorized under the PD-DM subtype. This is partially consistent with prior studies on the early drug-naïve PD-PIGD motor subtype at baseline, which have reported FA changes in the bilateral superior longitudinal fasciculi (SLF), bilateral anterior corona radiata (ACR), and CC, correlated with PIGD scores in PD patients compared to HCs (Maetzler et al., 2016; Minett et al., 2018; Yang et al., 2023). The CC contains crossing fibers that project to major bilateral cortical areas associated with motor and sensory functions (Hofer and Frahm, 2006). Dysfunction in the splenium and the posterior region of the CC' s body has been linked to gait impairments (Chan et al., 2014). In addition, the internal capsule and corona radiata are known to be related to movement dominance (Uhr et al., 2022). Therefore, we hypothesize that the pathogenesis of rigidity symptoms in patients with the PD-DM subtype may parallel that of the PD-PIGD motor subtype, with the CC potentially being a critical site of lesion (Simuni et al., 2016; Espay et al., 2017; Erro et al., 2019). This insight might also lay the groundwork for prognostic evaluation and targeted therapeutic interventions for patients with the PD-DM subtype presenting with rigidity symptoms.

Despite having comparable ages and disease durations, patients with the PD-DM subtype were more prone to exhibiting mild cognitive impairment, orthostatic hypotension (OH), and RBD at baseline (Fereshtehnejad et al., 2015; Erro et al., 2020). In an effort to elucidate the underlying pathological mechanisms of nonmotor symptoms in patients with the PD-DM subtype, we employed ICA to construct a DMN from the PD patient data. Our analysis revealed that the increased RD in the white matter region adjacent to the mPFC correlated significantly with the MOCA scores. In fMRI, the DMN is associated with internally oriented cognitive processes, including self-reflection, reminiscence, and prospective thinking, which emerge in the absence of external stimuli (Raichle, 2015). The mPFC, a central node within the DMN, plays a crucial role not only in episodic memory retrieval but also extends to other cognitive domains such as social cognition and emotional processing (Schilbach et al., 2008; Roy et al., 2012). Furthermore, from a neuropathological perspective, the presence of Lewy bodies, characterized by the accumulation of α-synuclein, in the neocortex—including the motor, prefrontal, temporal, and parietal cortices—corresponds to Braak stages 5 and 6 (Braak et al., 2003) when PD patients show classic cognitive deficits in clinical symptoms (Yousaf et al., 2018; Carceles-Cordon et al., 2023). Changes in RD may be attributed to the deposition of α-synuclein, which forms neuronal inclusions known as Lewy bodies (LBs) and Lewy neurites (LNs). This pathological process compromises the ability to restrict the diffusion of water molecules perpendicular to the axonal orientation, ultimately leading to demyelination and axonal injury in the adjacent white matter of the mPFC. The resulting microstructural damage to white matter is closely associated with the integrity of structural connectivity, which in turn has significant implications for cognitive function.

Bergamino et al. (2022) reported that FA values were more sensitive than MD values in evaluating the diagnostic efficiency of different parameters by using receiver operating characteristic (ROC) curves at baseline, and Minett et al. (2018) study demonstrated MD as a significant predictor of motor function decline in patients with early-stage PD at 1-year follow-up. We conjectured that this discrepancy may stem from the varying sensitivities of different DTI parameters to distinct phases of the disease. Our findings may validate a hypothesis that FA is a more robust composite metric for differentiating PD-DM subtype from other subtypes at baseline, while AD is a distinct metric for prognosticating disease progression.

At baseline and during the follow-up period, patients with the PD-DM subtype displayed a more severe decline in articulation compared to other subtypes, with a deterioration in semantic verbal fluency linked to elevated AD values. Probabilistic tractography of the region with elevated AD at baseline indicated that the area is primarily traversed by the SLF, arcuate fasciculus (AF), inferior longitudinal fasciculus (ILF), and inferior frontal-occipital fasciculus (IFOF). In contemporary dual-streams language network model, the ventral language stream is engaged in the semantic processing of speech, while the dorsal stream controls phonological processing (Binder and Desai, 2011). The AF and SLF are involved in the dorsal language stream, whereas the middle longitudinal (MLF), ILF, IFOF, and uncinate fasciculi (UF) are integral to the ventral language stream (Yagmurlu et al., 2016). Our result revealed that long-range connections emanating from the seed point participated in critical cortico-cortical pathways that constitute the language network. Future studies will quantify the association between these fiber tracts and cognitively relevant clinical scales. Additionally, our study also showed that increased AD in the superficial white matter (SWM) below the inferior parietal lobe (IPL), which is generally considered as the local connection present in the IPL (Zhang et al., 2021). IPL plays an important role in visuospatial and cognitive functioning, as well as in semantic processing networks. In this area, some fibers from the supramarginal gyrus and angular gyrus travel to the temporal and frontal lobes with the SLF, AF, and middle longitudinal fibers (Mdlf) (Burks et al., 2017). Together with ILF and IFOF, these tracts form a crossroad known as the temporal subcortical white matter/temporal-parietal fibers intersection area (TPFIA). In the past, this anatomy region has been pivotal for planning surgical strategies involving parietal and temporal strategies. This specific area stands out as especially vulnerable within the entire network. Even a minor lesion precisely located in this central neural hub can lead to extensive disconnections and significant consequences (Martino et al., 2013), which also provides a plausible explanation for the observed deterioration in semantic verbal fluency 1 year later caused by white matter damage in this region in PD-DM subtype.

Imbalances in the release of nigrostriatal dopaminergic neurons, along with functional and structural changes in the cortical striatal network, are important factors contributing to the clinical heterogeneity of PD subtypes. These factors also form the structural basis for the variability in the microstructure of white matter. In the early stages of disease progression, decreased putamenal dopamine uptake usually favors the side with the most severe symptoms (Mo et al., 2010). Our findings were consistent with those of previous studies, suggesting that the 123I-FP-CIT uptake in the left putamen was related not only to motor severity but also to SCOPA-ATU, such as OH (Simuni et al., 2018; Brücke and Brücke, 2021; Umehara et al., 2021). In addition, the putamen operates as a central integrative hub for diverse cortical inputs and is associated with an increased risk of dementia in PD patients. According to research by Chung et al. (2022), alterations within the cortico-striatal network, including a reduction in striatal dopamine levels and the disruption of the associated cortico-striatal circuits may significantly affect cognitive impairments that are contingent upon the integrity of the frontostriatal system in PD. In the present study, we have validated the predictive effect of diminished DAT uptake in left putamen at baseline on the decline of memory composite score (HVLT-total score) and MOCA score 1 year later. Furthermore, in patients with the PD-DM subtype, the pathological degeneration of dopaminergic neurons in the left putamen was associated with an increased RD in the frontal region, a pattern not observed in the other two subtypes. Cortico-striatal projections contain topographically organized fibers originating from the entire cerebral cortex. The basal ganglia receive input from the entire cerebral cortex, with a particular emphasis on the frontal lobes. The majority of afferent connections to the basal ganglia terminate in the neostriatal regions, specifically the caudate and putamen. The motor cortex of the frontal lobe projects to the centromedian nucleus and ends in the putamen, which plays a role in motor control. Posteriorly, neurons project from the thalamic nuclei to the motor cortex, premotor cortex, and prefrontal cortex (Leisman et al., 2014). In this circuit, the decrease in dopamine and acetylcholine released in PD patients affects synaptic plasticity, which in turn affects neuronal connectivity and function, and the decreased trophic action of nerves may lead to axonal damage or demyelination (Chu, 2020). Our findings revealed that pathological alterations caused by L-putamen dopaminergic depletion can induce motor and cognitive dysfunction through underlying neurodegeneration. This model contributed a deeper understanding of the pathological mechanisms of clinical impairment caused by white matter damage in patients with a new PD subtype, and these findings associated with the reduced DAT uptake in the left putamen in this study may offer new perspectives for clinical intervention strategies.

In our study among patients with the PD-DM subtype, we discovered a correlation between the decline in the levels of α-synuclein and β-amyloid in the CSF and a decrease in FA values, indicating that DTI is capable of detecting the effects of abnormal protein aggregation on axonal fibers. One of the possible pathological mechanisms is that Lewy neurites are the axonal manifestation of α-synuclein pathology and may be linked to impaired axonal transport, leading to structural alterations in the axon or its surrounding myelin (Picconi et al., 2012; Sharma and Burré, 2023). Our results are in line with prior research indicating that pathological deposition of α-synuclein and β-amyloid in the brain is accompanied by neuronal loss in various neurodegenerative diseases (Tokuda et al., 2006; Buddhala et al., 2015; Herholz et al., 2016; Zhang S. et al., 2023). Furthermore, Zhang et al., studying the association between cortical gyrification and imaging and serum biomarkers in PD, predicted that a reduction in axonal integrity, rather than gray matter atrophy, is the tissue pathological basis for the excessive reduction in cortical gyrification (Zhang Y. et al., 2023). Our findings support this perspective. In addition, the propagation of fibrillar α-synuclein from the brainstem to limbic and neocortical structures seems to be the most robust neuropathological indicator associated with the onset of dementia in PD patients (Braak et al., 2003; Carceles-Cordon et al., 2023). Moreover, up to 50% of PD patients with dementia also develop sufficient numbers of β-amyloid plaques and tau-containing neurofibrillary tangles, These pathological changes may interact synergistically with α-synuclein pathology, potentially contributing to a poorer prognosis (Irwin et al., 2013).

We conducted a whole-brain voxel-wise analysis on new clinical subtypes of PD and found that the white matter damage in the PD-DM subtype is partially consistent with the regions involved in the PD-specific network atrophy pattern. The PD-specific brain atrophy network evaluates major structures that are associated with either the functional connectivity (resting-state fMRI) or the anatomical connectivity (diffusion-weighted MRI) data originating from the substantia nigra, and the relative degree of atrophy in each brain region supports the theory of disease propagation through the connectome (Zeighami et al., 2015), which aligns with Braak's initial hypothesis (Braak et al., 2003). According to this hypothesis, the pathogenic forms of the α-synuclein protein spread from the substantia nigra throughout the nervous system, leading to a stepwise pattern of neurological impairment in PD (Braak et al., 2003; Del Tredici and Braak, 2020). This spread is believed to occur to neuronally connected but non-adjacent areas, with the accumulation of α-synuclein in axons potentially transforming into Lewy neurites, which can result in axonal damage and demyelination, preceding neuronal cell death (Masuda-Suzukake et al., 2013; Zeighami et al., 2015). Additionally, alterations in white matter integrity would precede adjacent gray matter atrophy (Masuda-Suzukake et al., 2013; Wolters et al., 2020). Fereshtehnejad et al. (2017) applied the PD-specific brain atrophy network map to compare each PD subtype with HCs and found that the degree of brain atrophy in patients with the PD-DM subtype is higher than in the other two subtypes, predominantly involved in the basal ganglia area, CC, premotor cortex, and occipital cortex. In our study, compared to HCs, patients with the PD-DM subtype showed decreased FA values in the CC, SLF, ILF, IFOF, and CST, which significantly correlated with α-synuclein levels in the CSF. The result of our study indicated that the disruption of intercortical and subcortical–subcortical structural connections may serve as the pathological basis for the specific brain atrophy network observed in patients with PD-DM subtype, thus providing theoretical support for the specific brain atrophy network observed in patients with PD-DM subtype.

Our study had several limitations. Firstly, while we accounted for LEDD as a covariate for all follow-up analyses, LEDD may increase until it is tolerated by the participant at 1-year follow-up, which may affect the clinical assessment of the patient. Secondly, due to the rigorous selection of patients, our sample size of PD-DM patients was relatively small, which could limit the generalizability of our findings and hence be a limitation of the study. Finally, although some participants withdrew from the study prior to the second MRI acquisition, there were no significant differences in demographic data and partly nonmotor scales between those who left and those who continued. However, the higher proportion of PD-DM patients with more severe motor symptoms among those who withdrew inevitably led to significant differences in motor-related scales (as detailed in Supplementary Table 2).

## Conclusion

This study presented a whole-brain voxel-level DTI analysis, aiming to integrate findings with SPECT and neurobiological insights to elucidate the underlying mechanisms of white matter microstructural damage and its progression in new clinical subtypes of PD. The current results showed that the symptoms of rigidity in patients with the PD-DM subtype followed a similar mechanism of white matter damage to the PD-PIGD subtype, with the corpus callosum being the key site. The damage in the white matter adjacent to the mPFC in patients with PD-DM was associated with cognitive impairment, which is consistent with the pathological manifestations of Braak stages 5 and 6, when α-synuclein in the CSF is deposited into the neocortex. Consequently, DTI features emerge as pivotal biomarkers for the early diagnosis of both motor and cognitive symptoms. These can facilitate the prompt identification of PD subtypes, thereby enabling the development of targeted therapeutic strategies tailored to the specific needs of different PD patient subtypes with the ultimate aim of enabling early intervention. Moreover, to some extent, DTI can assess overall disease progression and predict semantic functioning-related prognosis in de novo PD patients, providing a pathologic basis for evaluating long-term quality of life in patients with various subtypes of PD. In addition, stepwise deposition of α-synuclein, the imbalance of dopaminergic neuron release, and impaired function and structural connectivity of the cortico-striatal circuit further reveal that the patients with PD-DM subtype may follow a different pathological trajectory from the patients with the other two subtypes.

## Data availability statement

The datasets presented in this study can be found in online repositories. The names of the repository/repositories and accession number(s) can be found in the article/Supplementary material.

## Ethics statement

The studies involving humans were approved by Parkinson's Progression Markers Initiative Institutional Review Board. The studies were conducted in accordance with the local legislation and institutional requirements. The participants provided their written informed consent to participate in this study. Written informed consent was obtained from the individual(s) for the publication of any potentially identifiable images or data included in this article.

## Author contributions

XY: Writing – original draft, Writing – review & editing, Conceptualization, Data curation, Investigation, Methodology, Software, Supervision, Formal analysis, Funding acquisition, Project administration, Resources, Validation, Visualization. QY: Formal analysis, Funding acquisition, Project administration, Resources, Validation, Visualization, Writing – review & editing. YaL: Data curation, Formal analysis, Funding acquisition, Supervision, Validation, Visualization, Writing – review & editing. JC: Conceptualization, Methodology, Visualization, Writing – review & editing. JG: Formal analysis, Methodology, Project administration, Resources, Writing – review & editing. YuL: Data curation, Methodology, Software, Supervision, Writing – review & editing. RS: Data curation, Investigation, Resources, Writing – review & editing. YZ: Data curation, Formal analysis, Validation, Writing – review & editing. ZH: Conceptualization, Investigation, Writing – review & editing.
