# Supplementary material for: Microstructural alterations in white matter and related neurobiology based on the new clinical subtypes of Parkinson's disease
Source: Front Neurosci. 2024 Aug 1;18:1439443. doi: 10.3389/fnins.2024.1439443 (PMC11324559; doi:10.3389/fnins.2024.1439443)
Supplement: Supplementary file 1 [file Data_Sheet_1.docx]

**White Matter Microstructural Alterations and Related Neurobiology Based on the New Clinical Subtypes of Parkinson's disease**

**Classification criteria for new clinical subtypes of PD**

The scales included in the classification criteria for PD new clinical subtypes are as follows: Motor component: MDS-UPDRS II scale, MDS-UPDRS III scale, and Postural Instability and Gait Disorder (PIGD) score (calculated by the mean values for the NP2WALK and NP2FREZ parts of the MDS-UPDRS II scale and the NP3GAIT and NP3FRZGT and NP3PSTBL parts of the MDS-UPDRS III scale). Nonmotor components consists of 3 domains. Domain1: REM sleep behavior disorder (RBD) questionnaire. Domain2: Scales for Outcome in Parkinson’ s Disease -Autonomic (SCOPA-ATU) questionnaire. Domain3: Benton Judgment of Line Orientation scale (adjusted score), Hopkins verbal learning test-revised (HVLT) scales included HVLT-total recall (T-score), HVLT-delayed recall (T-score), HVLT-retention (T-score), and HVLT-recognition discrimination (T-score), Schwab and England activities of Daily Living (S&E-ADL) scale, Letter number sequencing (LNS) scale, Semantic fluency scale (T-score), and Symbol digit test scale (T-score)[[1](#_ENREF_1)].

**Probabilistic tractography steps**

At baseline, PD-DM showed increased AD values in the subcortical white matter of the right temporal-parietal fibers intersection area (TPFIA) compared with HC, indicated white matter damage. In order to track the white matter fibers connecting to the cortical regions. We extracted this cluster as the seeding region, Binarized and aligned to standard space (MNI152_brain_1mm) in fsl, Tractography began with within-voxel modeling of multi-fiber tract orientation structure via the bedpostx tool, and was followed by probabilistic tractography using probtractx2[[2](#_ENREF_2)]. Streamlines were seeded from each voxel in the cluster, and in order to tract all possible streamlines, we did not set the exclusion mask and termination mask. The default 0.5 mm step length, 5,000 samples and 2,000 steps were used. To avoid artifactual loops, streamlines that loop back on themselves were discarded (loopcheck). For each subject, the tracked streamline was normalized by the total number of generated streamlines (“waytotal” number). A threshold of 1% was used to binarize the probabilistic tractography to avoid false-positive streamline[[3](#_ENREF_3)]. Result shows in supplement material Figure [1](#fig1). Regions were located by mapping the statistical map to the John Hopkins University DTI white matter atlas within the FSL. the right association fibers (superior and inferior longitudinal fasciculus and inferior frontal-occipital fasciculus) and projection fibers (corticospinal fasciculus, posterior limb of the internal capsule, and superior and posterior corona radiata), splenium of corpus callosum and cingulate gyrus were observed[[4](#_ENREF_4)].

**Supplement material Fig. 1** The result of Protrack in PD patients


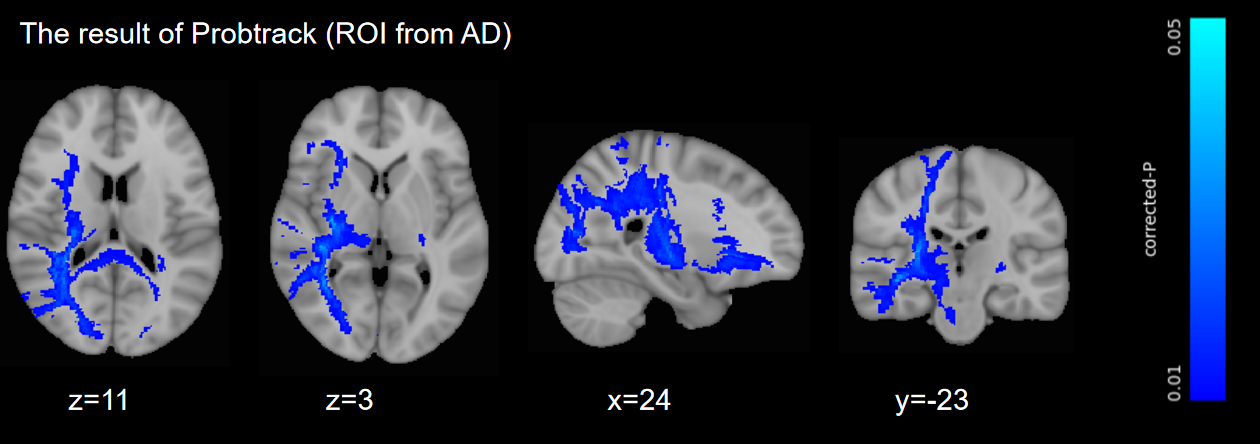


Supplement material Fig. 1 demonstrated that the results obtained after probabilistic fiber tracking using the region of difference in AD values of PD-DM patients in the body of the text as the seed point.

1. Fereshtehnejad S-M, Zeighami Y, Dagher A, Postuma RB, Clinical criteria for subtyping Parkinson’s disease: biomarkers and longitudinal progression. *Brain* **2017,** *140* (7), 1959-1976.<http://dx.doi.org/10.1093/brain/awx118>

2. Behrens TEJ, Berg HJ, Jbabdi S, Rushworth MFS, Woolrich MW, Probabilistic diffusion tractography with multiple fibre orientations: What can we gain? *NeuroImage* **2007,** *34* (1), 144-155.<http://dx.doi.org/10.1016/j.neuroimage.2006.09.018>

3. Tian Q, Wintermark M, Jeffrey Elias W, Ghanouni P, Halpern CH, Henderson JM, Huss DS, Goubran M, Thaler C, Airan R *et al*, Diffusion MRI tractography for improved transcranial MRI-guided focused ultrasound thalamotomy targeting for essential tremor. *NeuroImage: Clinical* **2018,** *19*, 572-580.<http://dx.doi.org/10.1016/j.nicl.2018.05.010>

4. Chu H-Y, Synaptic and cellular plasticity in Parkinson’s disease. *Acta Pharmacologica Sinica* **2020,** *41* (4), 447-452.<http://dx.doi.org/10.1038/s41401-020-0371-0>

| **Supplementary material Table 1** Differences in PD-DM and HC in men 1 year later | | | |
| --- | --- | --- | --- |
| Characteristic | PD-DM (12 cases male) | HC (23 cases male) | p value |
| age(years) | 63.67(9.1) | 67.01(9.3) | 0.323 |
| relationship of PD family | 0.46(0.5) | 0.09(0.2) | **0.029** |
| educational time(years) | 16.17(2.6) | 16.07(2.4) | 0.945 |
| scanning interval | 1.05(0.1) | 1.07(0.1) | 0.551 |
| FA value^a^ | 0.59(0.2） | 0.60(0.2) | **0.035** |
| AD value^a^ | 0.00127(0.00005) | 0.00121(0.00005) | **0.011** |
| RD value^a^ | 0.0004(0.000006） | 0.00039(0.0006） | 0.345 |
| After 1 year of follow-up, 12 males in PD-DM and 23 males in HC were analyzed for significance in the FA, AD, and RD regions of difference, and our results were consistent with the above follow-up results. a：ANCOVA used analysis, adjusted age, relationship of family, educational time(years) and scanning interval(years)**.** | | | |
|  |  |  |  |
|  |  |  |  |
|  |  |  |  |

| **Supplementary material Table 2** Demographic differences in left and continued participants   \| Characteristic \| left (56 cases) \| continued (124 cases) \| p value \| \| --- \| --- \| --- \| --- \| \| Female % \| 34.45% \| 50.00% \| 0.057 \| \| age \| 61.9 1(9.2) \| 61.75 (9.7) \| 0.916 \| \| educational time(years) \| 15.45(2.6) \| 15.82(3.4) \| 0.208 \| \| relationship of PD family \| 0.23(0.5) \| 0.38(0.2) \| 0.521 \| \| MDS-UPDRS II \| 6.02(5.1) \| 3.73(2.9) \| **0.029** \| \| MDS-UPDRS III \| 17.23(10.3) \| 11.66(10.7) \| **0.006** \| \| PIGD \| 0.11(0.19) \| 0.31(0.4) \| **0.004** \| \| MOCA \| 28.30(2.1) \| 27.81(1.7) \| 0.125 \| \| Semantic fluency \| 53.67(9.3) \| 52.72(9.8) \| 0.611 \|   56 cases left populations and 124 cases continued populations (included PD and HC) were analyzed in longitudinal cohort. There was no significant difference between 2 groups in terms of age、sex、educational time(years) and relationship of PD family. Because of the large proportion of PD-DM with more severe motor symptoms in the population that left the study, our motor-related scales demonstrated significant differences between left and continued. While representative non-motor symptoms: MOCA and Semantic fluency were not significantly different between the two groups. |
| --- | --- | --- | --- | --- | --- | --- | --- | --- | --- | --- | --- | --- | --- | --- | --- | --- | --- | --- | --- | --- | --- | --- | --- | --- | --- | --- | --- | --- | --- | --- | --- | --- | --- | --- | --- | --- | --- | --- | --- | --- |
